# Precision Beyond Limits: A Case Report of the Castable Resin Prosthesis

**DOI:** 10.7759/cureus.77165

**Published:** 2025-01-08

**Authors:** Angel Rose A., M. Saravanan, B. Muthukumar

**Affiliations:** 1 Prosthodontics, SRM Dental College, Chennai, IND

**Keywords:** cad cam, digital dentistry, fixed partial dentures, impression, wax pattern

## Abstract

This paper explores the application of castable resin in fabricating fixed partial dentures (FPDs), presenting a comprehensive companion to the fashion. Castable resin offers unknown perfection and customization, revolutionizing the field of prosthodontics. Despite its advantages, challenges like material selection and technological integration must be navigated. Future developments should improve the clinical mileage and case concerns of castable resin FPDs, effectively and perfectly altering the field of dental prostheses.

## Introduction

The advent of technology has caused various changes in dentistry to provide more efficient and effective treatments for people who require rehabilitation [1]. Traditional fabrication processes have developed as technology has advanced, with one example being the use of castable resin [2]. This article discusses castable resin prostheses' qualities, benefits, and uses, highlighting their transformative potential in the field. 

In the ever-changing environment of modern dentistry sophisticated materials and processes have transformed the process of making fixed partial dentures (FPDs). Among these advancements, castable resin has emerged as a game changer, providing unprecedented precision and diversity in prosthesis restoration. This article is a comprehensive guide that outlines the step-by-step process for creating FPDs with castable resin.

In this case, we present a patient where castable resin was used to design and fabricate the prostheses entirely through digital methods.

## Case presentation

**Figure 1:**
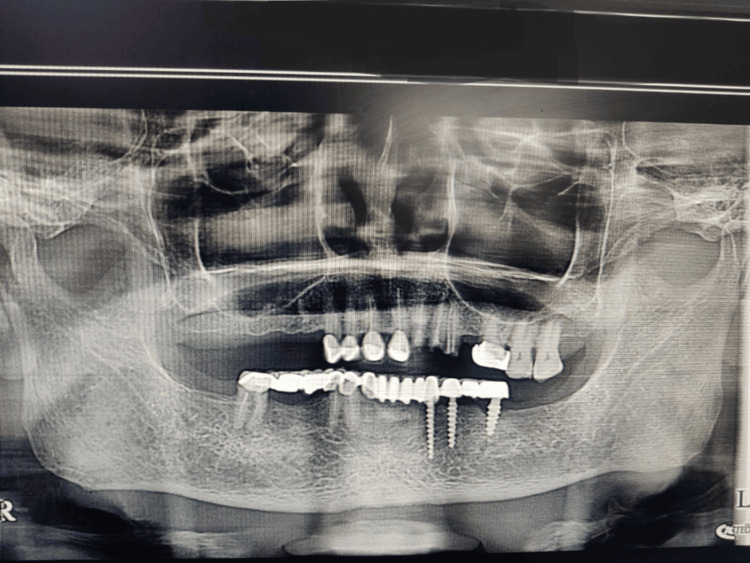
Preoperative OPG of the patient before the removal of the prosthesis.

A 71-year-old male patient reported to the Department of Prosthodontics and Crown and Bridge with a dislodged prosthesis in the complete lower arch and missing teeth in the upper arch in 14, 15, 16, 17, 22, 23, 24, and 25. The teeth in the lower arch included 33, 34, 36, 43, 44, and 46, with implants placed in positions 33, 34, and 36. The patient's preoperative OPG before removal of the prosthesis was taken (Figure 1). The prosthesis in the lower arch was removed.

Digital design

**Figure 2:**
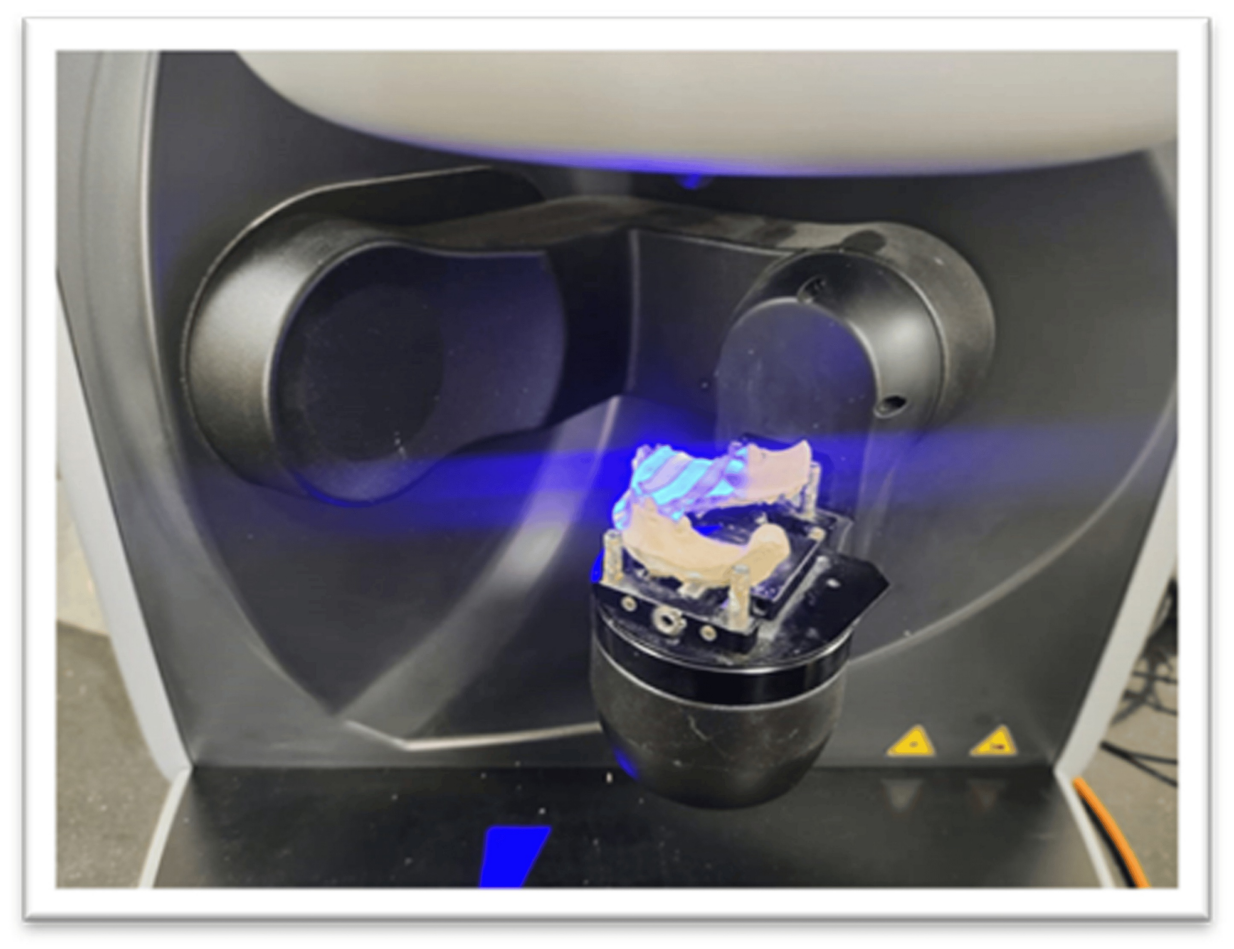
Scanning of the master cast using the Sirona EOS X5 extraoral laboratory scanner.

**Figure 3:**
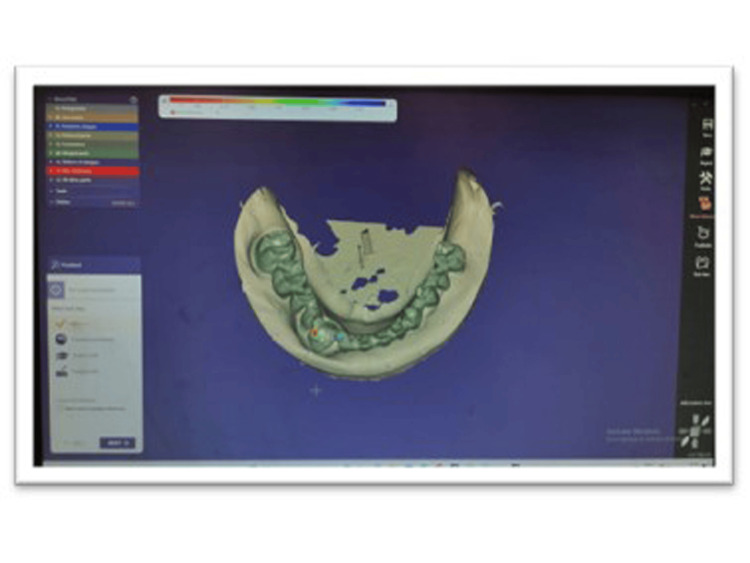
Digital design of the framework using Exocad software (Exocad GmbH, Darmstadt, Germany) and Meshmixer design software (San Rafael, CA).

Impressions of the patient were taken using polyvinyl siloxane impression material, and a cast was poured. The master cast was scanned using the Sirona EOS X5 extra-oral laboratory scanner (Figure 2). Computer-aided design (CAD) tools were then used to design the FPDs, ensuring optimal fit, occlusion, and appearance (Figure 3). Anatomical characteristics and functional requirements were incorporated into the digital design to achieve optimal outcomes in terms of precise marginal fit, aesthetics, and more. In this case, a key and keyway mechanism was included as an attachment in the anterior region, serving as a non-rigid connector between the natural tooth and the implant to prevent unnecessary biomechanical complications. These designs are easily created digitally using Exocad software (Exocad GmbH, Darmstadt, Germany) and Meshmixer design software (San Rafael, CA) [3].

Printing the framework

**Figure 4:**
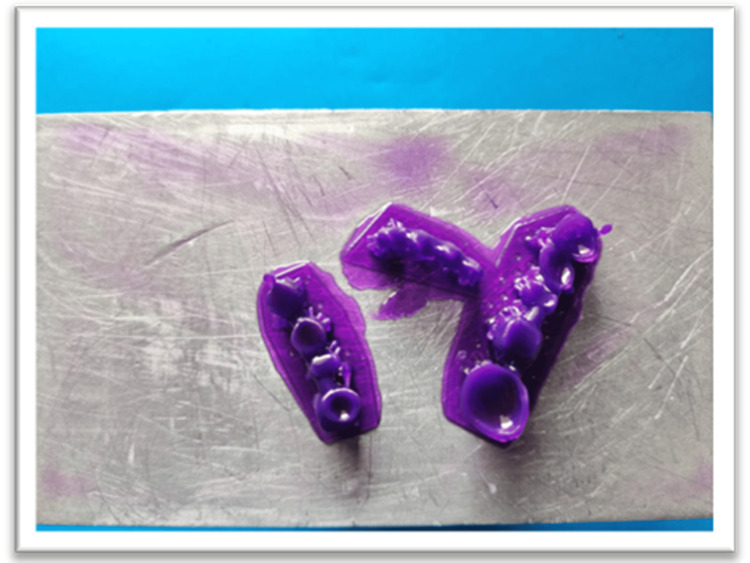
3D-printed framework using castable resin and the SLA method of 3D printing. SLA, stereolithography

**Figure 5:**
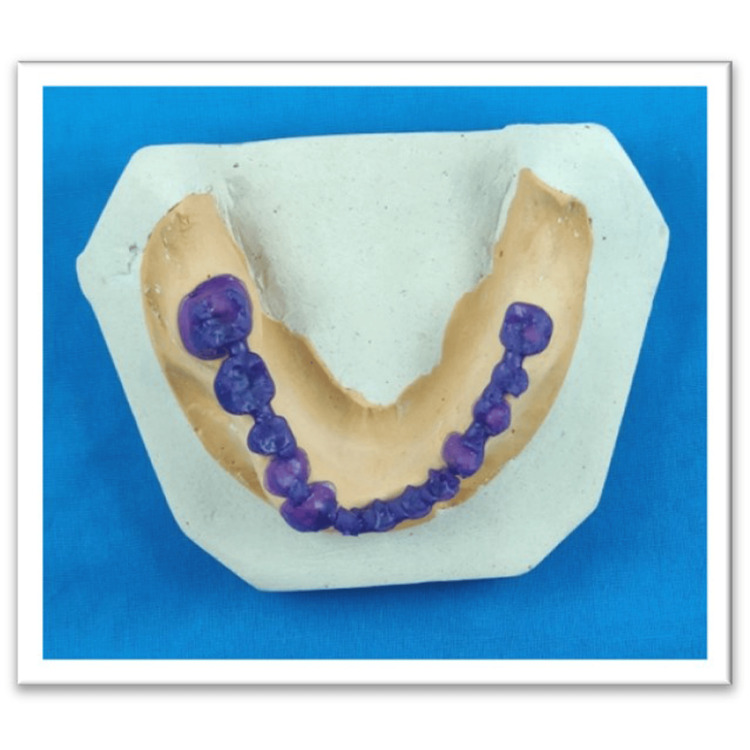
The resin curing unit framework is post-cured, fitted into the cast, and then used as a wax pattern.

The digital design was transferred to the 3D printer (Anycubic Photon Ultra, Anycubic, Shenzhen, China), and the relevant printing parameters were set (layer thickness: 0.05 mm per layer; curing time: 6.4 seconds per layer). The FPD framework was printed with castable resin to ensure high precision and accuracy (Figure 4). Before proceeding to the next stage, the printed framework was inspected for any faults or irregularities. The supports used for proper printing were removed with dental Tungsten Carbide burs, and the prosthesis was post-cured using the Anycubic Wash and Cure 3 (post-curing unit) (Figure 5). This served as the wax pattern [4,5]. 

Resin casting

The sprues were attached to the framework and silicone casting ring. The investment material was prepared according to the manufacturer's directions, ensuring proper mixing and degassing. The investment was carefully poured into the silicone casting ring, ensuring total coverage and proper adaptation to the framework, and the casting was then completed [6].

Finishing and polishing

**Figure 6:**
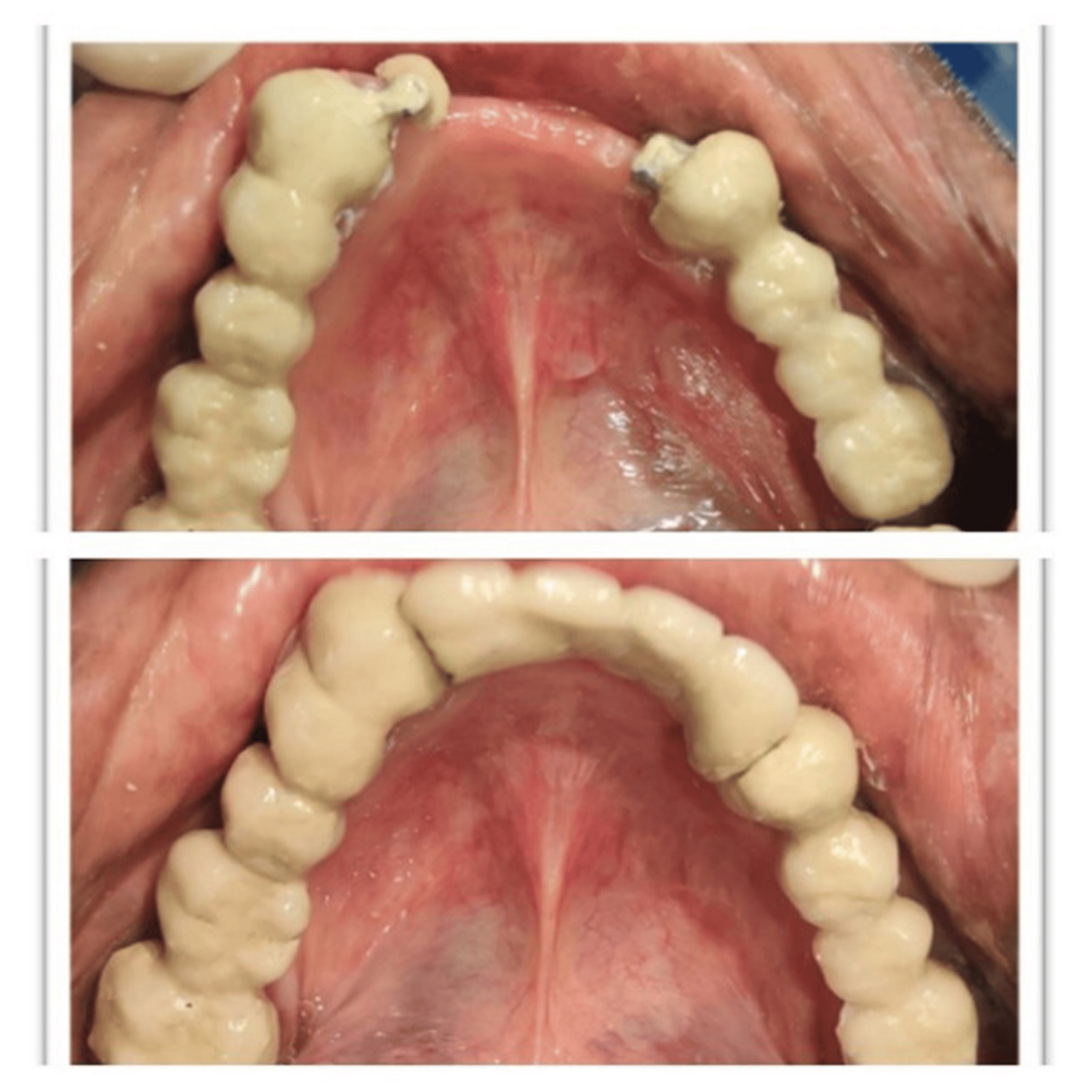
Bisque try-in of the completed prosthesis.

**Figure 7:**
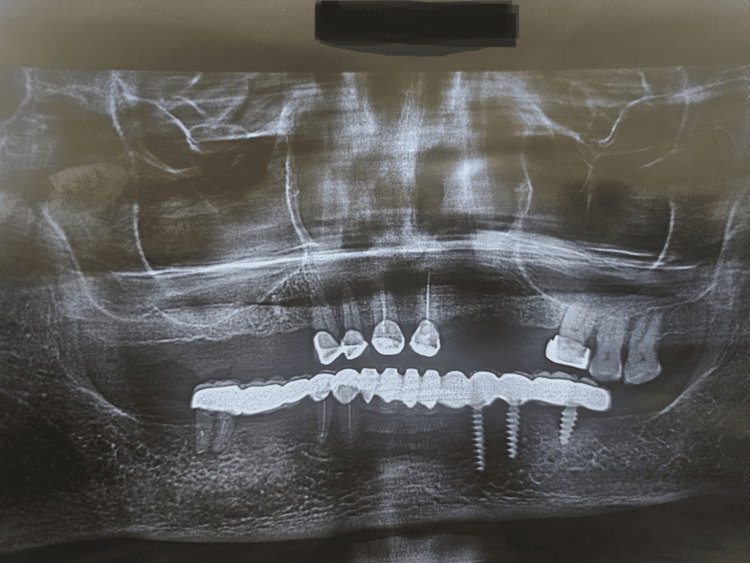
Post-insertion orthopantomogram (OPG) of the patient.

The FPD was polished with progressively finer grits of polishing compounds to achieve a smooth and shiny surface finish. A bisque try-in was performed (Figure 6), during which the fit and occlusion of the FPD were examined intraorally. Any necessary adjustments were made to ensure maximum comfort and function. Glass ionomer cement Type 1 (GC Gold Label 1 Luting and Lining GIC) was used to lute the prosthesis [7,8]. In the maxillary arch, a flexible partial denture was placed. A post-operative OPG was taken (Figure 7).

Finalization and delivery

The final FPD was disinfected using proper dental disinfectants to ensure patient safety. The patient was given appropriate oral hygiene instructions and maintenance advice for their new FPD. Follow-up appointments were scheduled to assess the FPD's performance and longevity, and to address any issues or concerns that might arise. The first recall appointment was scheduled one week after cementation, with follow-up visits advised every three months thereafter [9].

## Discussion

The integration of castable resin into the fabrication of FPDs represents a groundbreaking advancement in prosthodontics, leveraging the precision and versatility of high-resolution 3D printing alongside the customization potential of digital design. This approach allows for the creation of highly personalized prosthetic solutions that ensure a precise fit, optimal function, and superior aesthetics tailored to the unique anatomical and clinical needs of each patient. Furthermore, the efficiency of the digital workflow streamlines the production process, reducing the time and costs traditionally associated with manual methods, such as wax modeling and casting. This not only enhances clinical efficiency but also broadens the horizons of prosthetic innovation, enabling the design of intricate structures that were previously unattainable using conventional techniques.

However, the adoption of this technology is not without its challenges. A significant barrier lies in the initial investment required for digital tools, including 3D printers, specialized software, and high-quality resins, which can be prohibitive for smaller dental practices. Additionally, practitioners must undergo extensive training to master the use of CAD software and 3D printing technologies, which may entail a steep learning curve. Another critical consideration is the selection and development of suitable resins. Studies by Ambika compared the PLA and castable wax resin material models under compressive loading conditions. They revealed distinct mechanical behaviors. Wax exhibits higher von Mises stress and displacement, indicating greater deformation under compression when compared to castable wax resin [10]. Studies by Piangsuk et al. concluded that castable wax resin and castable resin exhibited comparable accuracy when post and core were fabricated using the 3D printing technique [11].

Achieving consistent and reliable outcomes also requires strict quality control throughout the fabrication process, from the digital design phase to the final production of the prosthetic device. The complexity of integrating emerging technologies like artificial intelligence (AI) further complicates the adoption process but simultaneously offers exciting opportunities. AI can enhance prosthetic fabrication by enabling predictive modeling, automated adjustments, and heightened customization, thus optimizing the accuracy and functionality of FPDs. This integration aligns with the overarching shift toward patient-centered care, fostering greater patient involvement in the design process and ensuring that prosthetic solutions are finely tuned to individual preferences and requirements.

Looking ahead, the potential of castable resin in prosthodontics continues to expand as advancements in material science and digital technology progress. The development of resins with improved mechanical and aesthetic properties will address existing limitations, paving the way for broader applications and more durable solutions. Furthermore, as AI and machine learning become more sophisticated, their role in enhancing workflow efficiency and clinical outcomes will grow, reinforcing the transformative impact of this technology. While challenges remain in terms of cost, training, and regulatory compliance, the benefits of castable resin-spanning improved efficiency, innovation, and patient satisfaction underscore its potential to redefine prosthodontic practices and elevate the standard of care in the field [12].

## Conclusions

Creating FPDs with castable resin demands precision, knowledge, uniform thickness, and attention to detail. By following this comprehensive guide, dental practitioners may maximize the revolutionary potential of castable resin technology to provide superior prosthetic solutions that improve patient results and happiness. As digital dentistry advances, the use of sophisticated materials and processes such as castable resin has the potential to change the future of prosthodontics by providing unparalleled precision, efficiency, and aesthetics in FPD production.
